# Rapid Adjustments in Thermal Tolerance and the Metabolome to Daily Environmental Changes – A Field Study on the Arctic Seed Bug *Nysius groenlandicus*

**DOI:** 10.3389/fphys.2022.818485

**Published:** 2022-02-16

**Authors:** Natasja Krog Noer, Mathias Hamann Sørensen, Hervé Colinet, David Renault, Simon Bahrndorff, Torsten Nygaard Kristensen

**Affiliations:** ^1^Department of Chemistry and Bioscience, Aalborg University, Aalborg, Denmark; ^2^UMR 6553, CNRS, Ecosystèmes, Biodiversité, Évolution, University of Rennes 1, Rennes, France; ^3^Institut Universitaire de France, Paris, France

**Keywords:** arctic, climate change, diurnal environmental variation, GC-MS metabolomics, insects, phenotypic plasticity, temperature variation, thermal tolerance

## Abstract

Laboratory investigations on terrestrial model-species, typically of temperate origin, have demonstrated that terrestrial ectotherms can cope with daily temperature variations through rapid hardening responses. However, few studies have investigated this ability and its physiological basis in the field. Especially in polar regions, where the temporal and spatial temperature variations can be extreme, are hardening responses expected to be important. Here, we examined diurnal adjustments in heat and cold tolerance in the Greenlandic seed bug *Nysius groenlandicus* by collecting individuals for thermal assessment at different time points within and across days. We found a significant correlation between observed heat or cold tolerance and the ambient microhabitat temperatures at the time of capture, indicating that *N. groenlandicus* continuously and within short time-windows respond physiologically to thermal changes and/or other environmental variables in their microhabitats. Secondly, we assessed underlying metabolomic fingerprints using GC-MS metabolomics in a subset of individuals collected during days with either low or high temperature variation. Concentrations of metabolites, including sugars, polyols, and free amino acids varied significantly with time of collection. For instance, we detected elevated sugar levels in animals caught at the lowest daily field temperatures. Polyol concentrations were lower in individuals collected in the morning and evening and higher at midday and afternoon, possibly reflecting changes in temperature. Additionally, changes in concentrations of metabolites associated with energetic metabolism were observed across collection times. Our findings suggest that in these extreme polar environments hardening responses are marked and likely play a crucial role for coping with microhabitat temperature variation on a daily scale, and that metabolite levels are actively altered on a daily basis.

## Introduction

Terrestrial ectotherms are subject to large spatial and temporal variability in their thermal environment (Kearney and Porter, 2009). In terrestrial ecosystems, daily changes in temperature can be substantial, and vary greatly with microhabitat characteristics such as topography and orientation, vegetation cover, shading and more (Sears et al., 2019; Kearney et al., 2020; Lembrechts and Lenoir, 2020). Some of the most extreme environments are found in the polar regions where the winters are long and cold, and the summers short and periodically hot (Bahrndorff et al., 2021b). During the arctic summer, daily temperatures can vary by >30°C and reach subzero temperatures at night (Convey et al., 2018; Davey et al., 2021). Organisms, including insects, living in these environments must therefore be able to survive and reproduce over a wide range of temperatures (Deere et al., 2006; Bahrndorff et al., 2021a). This can be achieved by evolutionary adaptation to the local thermal conditions across generations, or by fast adjustments of the physiology within the lifetime of an organism *via* phenotypic plasticity (Scheiner, 1993; Fusco and Minelli, 2010; Kristensen et al., 2020). Evolutionary adaptation to changing and periodically stressful temperatures can be slow, and are sometimes constrained by genetic trade-offs or lack of adaptive genetic variation (Araújo et al., 2013; Hoffmann et al., 2013). Conversely, rapid plastic adaptive changes can rescue individuals exposed to biotic and abiotic challenges at a shorter timescale, including daily environmental fluctuations (Colinet and Hoffmann, 2012; Noer et al., 2022). Plastic changes might therefore be particularly relevant for arctic species exposed to unpredictable and rapid changes in the environment.

Organisms can respond plastically to short-term exposure to sub-optimal temperatures through hardening or by acclimation at longer term exposures (Colinet and Hoffmann, 2012; Schou et al., 2017). Hardening responses to extreme or acute temperatures are thought to counter rapid thermal stress, such as daily temperature extremes and stochastic events (Koveos, 2001; Hoffmann et al., 2003; Kelty, 2007; Overgaard and Sørensen, 2008; Jensen et al., 2019). The other form of more gradual acclimation includes seasonal acquisition of cold or heat tolerance that is induced by changes in temperature and photoperiod interacting with other abiotic factors (reviewed by Chown and Terblanche, 2006; Teets and Denlinger, 2013). There are several published examples of cold acclimation and rapid cold hardening in arctic arthropods (e.g., Bahrndorff et al., 2007; Everatt et al., 2013), but very few studies have investigated physiological acclimation of polar terrestrial arthropods to high temperatures (Sørensen et al., 2019; Bahrndorff et al., 2021b). Traditionally, thermal plasticity of insects has been investigated using model-organisms kept and hardened/acclimated to constant controlled temperatures in the laboratory (Angilletta, 2009; Colinet et al., 2015; Javal et al., 2016; Ketola and Kristensen, 2017). However, recent work on the impacts of temperature variability on thermal tolerance have emphasized that thermal performance based on constant temperatures do not always accurately predict performance under variable conditions in the laboratory (reviewed by Colinet et al., 2015; Vázquez et al., 2017), nor in the field (see e.g., Kingsolver and Nagle, 2007; Loeschcke and Hoffmann, 2007; Kristensen et al., 2008; Ketola and Kristensen, 2017). This potential mismatch in the conclusions arising from investigations based on constant *versus* fluctuating temperatures partly results from the non-linear impact of temperatures on thermal performance (Jensen’s inequality) (Ruel and Ayres, 1999; Colinet et al., 2015), time-by-temperature interactions (Foray et al., 2013; Kingsolver et al., 2015), and methodology (Chown et al., 2009; Mitchell and Hoffmann, 2010; Bahrndorff et al., 2016). Based on such results, the potential for transferring the knowledge obtained from the laboratory to field conditions, and thus forecast reliable predictions of the effects of climate change on the responses and geographic distribution of insects, is being increasingly questioned (Fischer et al., 2011; Kingsolver et al., 2015; Kinzner et al., 2019; Taylor et al., 2021).

The physiological and molecular mechanisms enabling arthropods to tolerate temperature stress has previously focused on controlled laboratory studies (but see Tomanek and Somero, 1999, 2002; Buckley et al., 2001; Gracey et al., 2008; Kristensen et al., 2012; Vasquez et al., 2019). Studies on temperate and polar species suggest (causation is typically lacking in such studies) that metabolites such as sugars, free amino acids and polyols can be associated with changes in cold tolerance measures (Zachariassen, 1985; Fields et al., 1998; Sømme, 1999; Holmstrup et al., 2002; Michaud and Denlinger, 2007, 2010; Overgaard et al., 2007, 2014). Changes in polyols on the other hand have been associated with changes in heat tolerance (Hendrix and Salvucci, 1998; Wolfe et al., 1998; Salvucci et al., 2000; Benoit et al., 2009). However, few have attempted to describe how these metabolites are affected by dynamic and fluctuating temperatures as encountered in nature (but see Kristensen et al., 2012; Noer et al., 2020; Sheldon et al., 2020).

In this study, we examined the effects of daily variation in the microhabitat temperatures on plastic adjustments of heat and cold tolerance of the Greenlandic seed bug *Nysius groenlandicus* (Zetterstedt) during summer in Southern Greenland. *Nysius groenlandicus* is a univoltine species, and widespread and abundant in Arctic and sub-Arctic regions. Previous work on the species have revealed that it can rapidly increase heat tolerance when exposed to high and stressful temperatures under laboratory conditions (Sørensen et al., 2019), and thus *N. groenlandicus* represents a valuable polar insect model for field-based description of daily changes in individuals’ thermal tolerance. Further, we examined the metabolic fingerprints within days with high and low temperature variation using a quantitative targeted gas chromatography-mass spectrometry (GC-MS) approach. We hypothesized that the ability to tolerate low and high temperatures is constantly fine-tuned to respond to temporally fluctuating temperatures in field-collected individuals of *N. groenlandicus* as an adaptation to the highly variable environmental conditions within and across days. Thus, we expected specimens collected in early morning and late evening to have the highest cold tolerance, while those collected at midday exhibiting the highest heat tolerance. Finally, we expected to see daily changes in metabolites known to improve cold tolerance (sugars and amino acids) in individuals collected in early morning and late evening, while metabolites enhancing heat tolerance (polyols) would show higher concentrations in individuals sampled during the warm periods of the day.

## Materials and Methods

### Field Collection and Experimental Design

**FIGURE 1:**
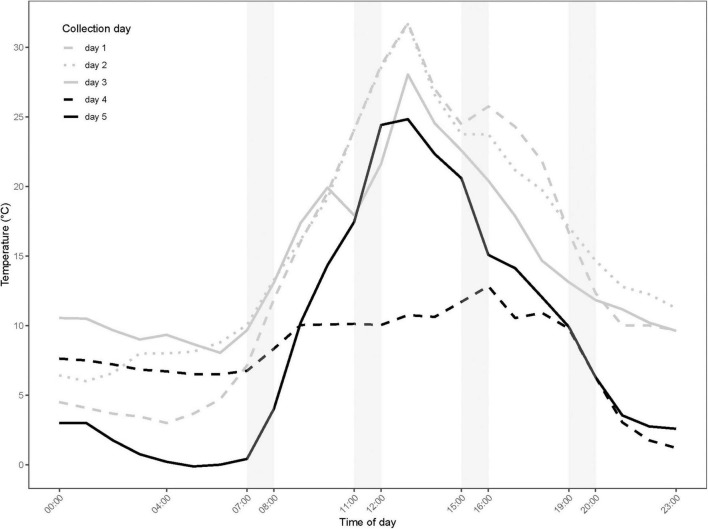
Microhabitat temperature (°C) averaged in 1-h bins across the five experimental days. The temperatures were measured at 15 cm above the soil surface in the shade every 5 min. Samples for GC-MS analysis were collected on day 4–5 (black lines). Shaded columns indicate the timespans where specimen are collected.

The field work was conducted in July–August 2018 in Narsarsuaq (Southern Greenland, 61.160°N, 45.424°W). This region is characterized by cool temperatures, long winters and short and thermally variable summers (Böcher and Nachman, 2001; Bahrndorff et al., 2021b). The study site was a heath-like, grass-covered area, where adult *N. groenlandicus* were collected from the grasses using a sweep net (Figure 1). All individuals used in the experiment were caught in a 50 m × 50 m area less than 100 m from the laboratory facilities.

Adult individuals of *N. groenlandicus* were collected at four time points (08:00 am, 12:00 pm, 04:00 pm, and 08:00 pm) during each of 5 days (depending on weather conditions and the abundance of *N. groenlandicus*; see Supplementary Table 1 for exact collection times and dates), thereafter referred to as day 1 to day 5. At the time of field-collection, each individual was placed in a 4 mL screw-cap glass vial (45 mm × 14.7 mm) and placed in the shade on the ground to prevent abrupt changes in the thermal conditions. The sex of individuals was then assessed by eye, and the vials transferred to the laboratory within 30–45 min of collection. Immediately after returning, the heat and cold tolerances were scored using 20 females and 20 males for each assay (see next section).

Additional individuals were collected for subsequent metabolomics analysis at the same four sampling times at two dates (22/08/18 and 27/08/18, representing day 4 and 5, respectively, see Supplementary Table 1), representing days with either high or low observed temperature variation. At each collection time, eight samples of five females (only females were used for the metabolomics studies) were collected, transferred directly into ice cold RNAlater, and stored at −20°C for approximately 1 week. Then the samples were transferred to our laboratory in Denmark where they were stored at −80°C and later used for metabolomic fingerprinting. Collection of samples in RNAlater is amenable for downstream metabolomics analysis if stored at subzero temperatures (van Eijsden et al., 2013; Harris, 2018).

The air temperature at the collection site was continuously recorded with 5-min intervals in the shade using Easylog USB data loggers (LASCAR Electronics, EL-USB-2^+^). The temperature was measured in the shade to avoid warm temperature-spikes in the measurements caused by direct solar radiation (Maclean et al., 2021) and the loggers were placed 15 cm above the soil surface to reflect the thermal environment at the top of the grasses where *N. groenlandicus* was most abundant, and was caught with the sweeping net. Based on these recordings, the mean temperature was calculated for the 1-h timespan prior to testing thermal tolerances. The mean temperature immediately prior to thermal assessment has been shown to be highly correlated with the heat tolerance in a range of insect species collected in the field (Noer et al., 2022).

### Heat and Cold Tolerance Assays

#### Heat Knockdown Time

Heat tolerance was measured as heat knockdown time (HKDT), i.e., a measure of the time before individuals go into a heat-induced coma/die at a high stressful temperature (Terblanche et al., 2007; Bak et al., 2020). For *N. groenlandicus* HKDT constitutes a direct measure of heat tolerance as we register the time individuals can withstand before they die. The vials containing field-collected individuals were mounted to a rack and submerged into a temperature-controlled water bath (PolyScience MX Immersion Circulator: MX-CA12E) maintained at 48°C. The individuals were then observed and stimulated with flashes of light and gentle taps on the vial caps with a metal rod. The time until movement ceased was noted for each individual. The chosen HKDT temperature was based on experiences from previous work on the species (Sørensen et al., 2019), and on unpublished preliminary results showing that *N. groenlandicus* individuals went into coma within 20–40 min at 48°C. Earlier results suggest a fast heat hardening response for this species and since we wanted a measure of their acute heat tolerance, and aimed for reducing hardening responses induced while testing, this HKDT was found relevant.

#### Chill Coma Recovery

Cold tolerance was measured as the temperature at which the bugs regained the ability to move after being knocked down by exposure to a low temperature (T_recovery_) following a modified procedure of the method described in Overgaard et al. (2011). Thus, we used a proxy rather than direct measures of cold tolerance. The glass vials containing the individuals were mounted to a rack and submerged into a glycol-water solution that was kept at −3°C using a thermostat (LAUDA Proline Edition X RP 1845-C, LAUDA DR. R. WOBSER GMBH & CO., KG, Germany). This temperature was based on the lower critical temperature (CTmin) which induces a cold coma for the species (Bahrndorff et al., 2021b). Immediately after submersion, the temperature of the solution was increased at a rate of 0.2°C/min. Pilot studies showed that this temperature (−3°C) was sufficient to induce chill coma within a few minutes with full survival upon returning to room temperature (data not presented). Following the HKDT procedure, the individuals were observed and provoked using light flashes and gentle taps, and the temperature at which individuals first moved any body part was noted as their chill coma recovery temperature (T_recovery_). A low T_recovery_ is interpreted as high cold tolerance.

### Metabolomic Fingerprinting

We adapted the methods detailed in Thiébaut et al. (2020) for detecting and quantifying the metabolite content from whole-body extracts of female *N. groenlandicus*. For each field sampling time, eight replicates were used, each consisting of five pooled females to obtain sufficient biomass (∼4 mg dry mass per sample). Each sample was vacuum dried (Speed Vac Concentrator, miVac; Genevac Ltd., Ipswich, England) and weighed (Mettler Toledo UMX2, accurate to 0.001 mg) before extractions so that metabolite concentrations could be reported according to dry mass. The samples were first homogenized for 90 s with two tungsten beads in 450 μL of a solution of ice cold methanol-chloroform (ratio 2:1, v:v) using a bead beater (Qiagen MM301; Retsch GmbH, Haan, Germany) set at 25 Hz. To separate the homogenate in two distinct phases (lipid-rich containing phase, and aqueous phase containing metabolites), 300 μL of cold ultrapure water was added to each tube before centrifugation (Sigma 2-16K, Sigma GmbH, Harz, Germany) for 10 min at 4000 *g* at 4°C. The supernatants containing metabolites were transferred to new tubes and stored at −80°C until analysis by GC-MS. Prior to the analysis, 120 μL of the metabolite extract was transferred to glass vials and vacuum dried.

The derivatization of the samples (dry residues) was automatized with a CTC CombiPAL autosampler (CTC Analytics AG, Zwingen, Switzerland). Dried samples were re-suspended in 30 μL of 25 mg mL^–1^ methoxyamine-hydrochloride (CAS Number: 593-56-6, SIGMA-ALDRICH, St. Louis, MO, United States) in pyridine prior to incubation under orbital shaking at 40°C for 60 min. Following incubation, 30 μL of N-methyl-bis(trifluoroacetamine) (BSTFA, CAS Number: 685-27-8) was added, and derivatization was conducted at 40°C for 60 min under agitation. An Agilent 7890B gas chromatograph coupled to a 5977B mass spectrometer was used for the separation and detection of the metabolites. For each sample, 1 μL was injected (Injector temperature: 250°C; split mode with a split ratio of 25:1); the temperature of the oven increased from 70 to 170°C at a rate of 5°C/min, from 170 to 280°C at 7°C/min, and from 280 to 320°C at 15°C/min, and then remained at 320°C for 4 min. We used a 30-m fused silica column (HP5 MS 30 m, I.D. 0.25 mm, thickness 0.25 μm, 5% Diphenyl/95% Dimethylpolysiloxane, Agilent Technologies), and the gas carrier (Helium) had a flow of 1 mL per min. The temperatures of the transfer line and ion source were 280 and 230°C, respectively. Metabolite fragmentation and ionization were carried out by electronic impact (electron energy: 70 eV) and detected with the full scan mode. The detected peaks were identified and annotated with MassHunter (Agilent). Most detected metabolites were identified, and calibration curves were used with pure compounds for calculating the concentration of each metabolite.

### Statistical Analysis

Differences in thermal tolerance with sampling time were examined using two-way ANOVAs for male and female *N. groenlandicus* separately with “time to knockdown” or “chill coma recovery temperature” as dependent variables, and “time of day” and “day” as independent variables. The tests were run on individuals. To ensure normal distribution, the data were transformed using rank inverse transformation and the models were run on both transformed and non-transformed data for validation. In addition, we used Pearson’s correlations to examine if microhabitat temperature affected thermal tolerances rather than sampling time alone. The Pearson’s correlations were calculated using the mean field temperatures observed in the 1 h preceding each collection round and the mean HKDT and T_recovery_ for each assay and sex (*n* = 20).

We used one-way ANOVAs to examine if individual metabolite concentrations within each day differed according to collection time. The concentrations of all quantified metabolites were then scaled and mean centered. For each sampling day, between-class PCA (R-package “ade4”), were run to explore the daily temporal structure of the metabolomic profiles. Monte Carlo tests (1000 permutations) were used to examine the significance of differences in metabolite profiles among classes of individuals from the four sampling times. Further, the metabolites that contributed the most to separation of groups were identified and ranked by their correlation to the principal components that described most of the inertia in the data. All analyses were carried out using the software “R” (R Core Team, 2020). Raw temperature and GC-MS files used for the analyses are available in the Supplementary Material.

## Results

### Microhabitat Temperatures

Observed microhabitat temperatures of the different sampling periods are shown in Figure 1. The largest daily amplitude recorded was 28.8°C on day 1, while the lowest of 11.6°C was on day 4. The warmest average temperature in the 1-h timespan prior to thermal tolerance tests was 26.3°C (see Supplementary Table 1 for exact temperatures) and was recorded at midday on day 2; the coldest average temperature prior to tests was 1.0°C and was recorded in the morning of day 5.

The microhabitat temperatures varied markedly between the two experimental days where samples were collected for metabolomic profiling (day 4 and 5; Figure 1). Within day 4, the temperatures were relatively constant with only 4.9°C difference in the average temperatures at the four daily collection times. Day 4 was also characterized by the lowest recorded daily amplitude. Day 5 was characterized by a high temperature variation with an amplitude of 25°C; the average temperatures at the four collection times differed by 21°C (Figure 1 and Supplementary Table 1).

### Thermal Tolerance

**FIGURE 2:**
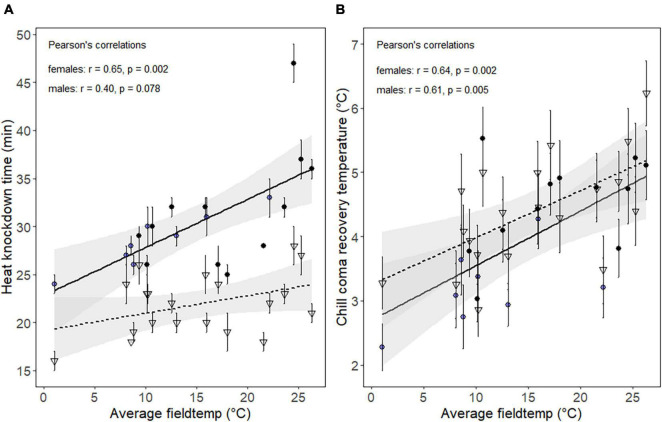
Scatter plot showing the mean **(A)** heat knockdown time in minutes, and **(B)** chill coma recovery temperature (T_recovery_) of females (circles) and males (triangles), as a function of the average microhabitat temperature 1 h prior to assay start. At eight sampling times, females were collected for metabolomic fingerprints simultaneously with individuals used for the thermal assays (blue circles). Black points are data points with no metabolomic fingerprints associated. Solid (female) and dotted (male) lines represent regressions between field temperature and thermal tolerances. Bars are standard errors of the mean.

Thermal tolerances varied significantly within and between days for both male and female individuals (Supplementary Figure 2 and ANOVA Supplementary Table 2). The average HKDT at each collection time and day was correlated with the average field temperature observed 1 h prior to heat tolerance assessment. The correlation was significant for females but not for males (Figure 2A). The regression slope, representing the change in HKDT per °C change in field temperature, was 0.36 min/°C for females and 0.18 min/°C for males. In addition, HKDT was overall higher for females than for males at any given field temperature with a difference of 5 min at the intercept. The maximal difference in mean HKDT measured across all temperatures was 23 min for females and 12 min for males. The relation between mean T_recovery_ and the average microhabitat temperature preceding cold assays revealed a significant positive relationship for both females and males (Figure 2B). The increase in recovery temperature per °C change in field temperature were 0.09 and 0.07°C for females and males, respectively. The largest difference in T_recovery_ found across all days was 3.3°C for females and 3.4°C for males.

For the days where females were collected for metabolomic fingerprinting, the HKDT and T_recovery_ varied markedly across the thermally variable day (day 5), and less so on the thermally stable day (day 4) (Supplementary Figure 2). Thus, the correlation between the field temperature and heat tolerance of the females collected only at the time points used for metabolomic profiling was strong and highly significant (*R* = 0.94, *p* = <0.001). The relationship between field temperature and T_recovery_ of females collected on day 4 and 5 was also positive and directional, however, not significant (*R* = 0.52, *p* = 0.19).

### Metabolomic Profiling

#### Description of the Quantified Metabolites in the Samples

The full scan monitoring allowed us to identify and quantify 33 metabolites with the quadrupole GC-MS platform (Supplementary Table 3; and see raw metabolite concentrations in Supplementary Table 4). We quantified 13 free amino acids, five sugars, six polyols, six metabolic intermediates, and three other metabolites. Xylitol and ethanolamine concentrations were below the quantification limit (i.e., below the lowest concentration of the calibration curve) in many samples and were therefore excluded from the quantitative profiling. The most abundant metabolites across all sampling times and days were phosphoric acid, proline, glutamate, and tyrosine.

#### Effects of Collection Time on Metabolic Fingerprints

**FIGURE 3:**
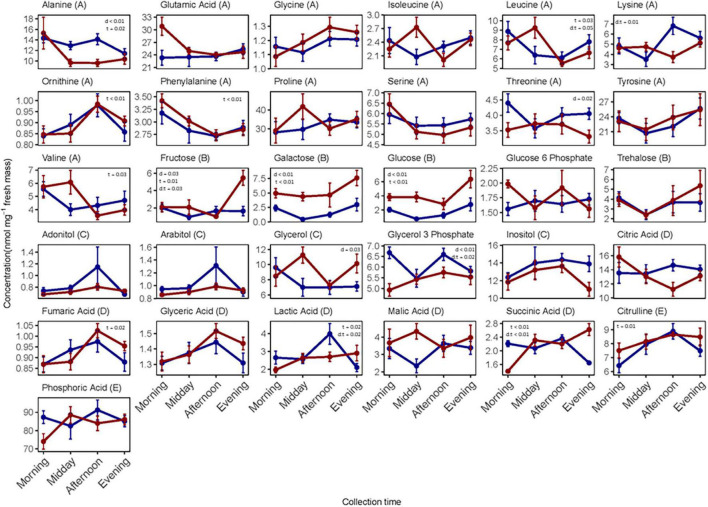
Individual metabolite concentrations (nmol mg^–1^ dry mass) measured in whole-body extracts of female *N. groenlandicus* collected from the field at four consecutive time points (morning, midday, afternoon, and evening) during day 4 (blue) and day 5 (red). Each point represent mean concentration (*n* = 8) and bars are standard errors of the mean. Differences between concentrations (log-transformed) and collection days (d), collection times (t), and the interaction between day and time (d:t) was investigated using 2-way ANOVAs and significance *p*-values are shown on each plot if significant. The metabolites are classified from A to E in the header according to functional group; A, amino acids; B, sugars; C, polyols; D, metabolic intermediates; and E, other metabolites.

Individual metabolite concentrations for each sampling time are displayed in Figure 3. We observed that a large number of metabolites varied in concentrations across collection times on day 5. There was a significant effect of “time of day” on the levels of eight metabolites (Supplementary Table 5). Conversely, the metabolite concentrations varied less on collection day 4, though there was a significant effect of “time of day” on concentration for seven of these (Supplementary Table 5). To examine whether the concentration levels varied similarly across collection times for the two collection days, we ran 2-way ANOVAs for each metabolite. Six metabolites varied distinctively across the four collection times for the 2 days, and these are depicted as the interaction between collection day and time on Figure 3. Finally, the sugars glucose, fructose, and galactose and the polyol glycerol occurred in larger concentrations in individuals collected on day 5.

**FIGURE 4:**
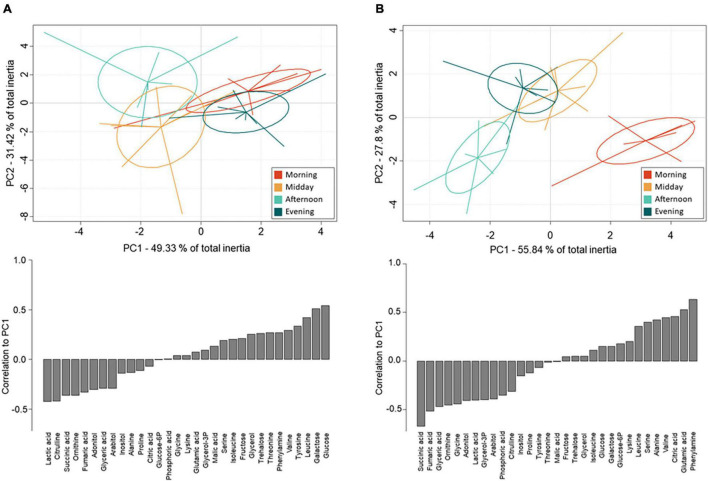
Between-class Principal Component Analyses (top) and metabolite correlations to PC1 (bottom) based on GC-MS quantification of metabolites from whole-body extracts of female *N. groenlandicus* sampled in the field at four consecutive sampling time points (8:00 am, 12:00 pm, 4:00 pm, 8:00 pm) on **(A)** the thermally stable sampling day (day 4) and **(B)** the thermally variable sampling day (day 5). Lines represents individual sample positions respective to centroids (*n* = 8).

Between-class PCAs were run separately on the metabolite concentrations from individuals sampled on day 4 (Figure 4A) and day 5 (Figure 4B). PC1 and PC2 cumulated 80 and 83% inertia on day 4 and 5, respectively; thus, on both days, the first two PCs explained most of the between-class variation. All classes showed a clear-cut separation meaning that metabolomic fingerprints differed among the different sampling times. The separation appeared stronger on day 5 (Figure 4B) than on day 4 (Figure 4A). Monte-Carlo randomization tests confirmed differences in metabolomic profiles among classes on day 5 (*p* < 0.001) and day 4 (*p* = 0.026) and the significance levels underpins that metabolomic profiles differed more markedly between sampling times on the thermally variable day 5 compared to on the less thermal variable day 4, as evidenced by much lower ellipses overlap.

#### Effects of Low Daily Thermal Fluctuations on Metabolic Fingerprints

On day 4, which was characterized by low temperature variation, the metabolomic profiles of individuals collected in the morning and evening differed from the profiles from midday and afternoon along PC1 (Figure 4A). Further, the metabolic fingerprints of individuals collected during midday and afternoon separated along PC2.

The metabolites most positively correlated to PC1 were the sugars galactose and glucose (Figure 4A). These two sugars were more abundant in individuals sampled in the morning and evening compared to those sampled at midday and afternoon. Lactic acid and citrulline were the metabolites that were most negatively correlated to PC1, and they were more abundant in individuals sampled at midday and afternoon. The polyol glycerol-3-phosphate and succinic acid had the highest positive associations with PC2 (Supplementary Figure 3) and were characterized by higher concentrations in individuals sampled in the morning and afternoon. Only one metabolite, glucose-6-phosphate, were negatively associated with PC2 and thus more abundant in the morning.

#### Effects of High Daily Thermal Fluctuations on Metabolic Fingerprints

On day 5, metabolomic profiles of individuals collected in the morning separated strongly from individuals sampled in the afternoon (Figure 4B). These two collection times were also the two thermal extremes of the day (i.e., the lowest and highest temperatures of the day, see Figure 1 and Supplementary Table 1); this observation suggests that PC1 explained metabolic changes correlated to high diel thermal variation. Further, the metabolic fingerprints of the individuals collected at these time points separated from the fingerprints of individuals collected at midday and in the evening along PC2.

The metabolites that were positively associated with PC1 on day 5 were phenylalanine, glutamic acid, citric acid, and to a lesser extent other amino acids, including valine, leucine, serine, and alanine (Figure 4B). These were more abundant in individuals sampled in the morning compared to individuals from the other collection times. The metabolites that were most negatively correlated to PC1 were succinic acid, fumaric acid, glyceric acid and ornithine. Further, negative correlations included several polyols (adonitol, glycerol-3-phosphate, arabitol) whose concentrations were higher in individuals collected in the afternoon than in the morning. The sugars fructose, glucose and galactose, and the amino acids isoleucine and lysine, as well as succinic acid and glycerol were positively associated with PC2 (Supplementary Figure 3), and were thus more abundant in individuals sampled in the evening. Negative associations were few, but the most negatively associated metabolite was the sugar glucose-6-phosphate which was more abundant in the morning and afternoon.

## Discussion

### Diel Variations in the Thermal Tolerance of Field-Sampled Insects

In our study, we showed a linear relationship between ambient microhabitat temperature and measures of both cold and heat tolerance of field-collected specimens of *N. groenlandicus*. Given the temperatures observed in the field during summer in Narsarsuaq includes subzero night temperatures and peak day temperatures above 40°C (Sørensen et al., 2019; Bahrndorff et al., 2021b) we advocate that the ability to withstand and remain active at high temperatures and recover fast from low temperature coma is ecologically important, especially for a univoltine species such as *N. groenlandicus*. This allows the species to, e.g., forage and mate in a transient environment and short summer season. Thus, our results point to plasticity in thermal tolerances being of strong importance for survival of insects in this region. Recently, the heat hardening capacity of *N. groenlandicus* has been examined in the laboratory, showing that heat tolerance can be increased within 45 min when the insects are exposed to high temperatures (Sørensen et al., 2019). It was also found that the heat hardening effect was reverted within 2 h when the insects were returned to cooler temperatures. Our results are consistent with these former observations, and the rapid adjustments that we observed in both heat and cold tolerance from field-sampled *N. groenlandicus* additionally support the assumption that hardening responses are important for coping with rapid changes in ambient temperature in the field. This is in contrast to the slower hardening responses observed in some temperate insect species and indicates that high thermal variability of the environment can be a selective agent for rapid plastic responses (Dahlgaard et al., 1998; Bahrndorff et al., 2009; Alemu et al., 2017).

Interestingly, the adjustments occurred not only at extreme temperatures, but also at temperatures that are not considered as stressful or sub-lethal to the species. The body temperatures experienced by *N. groenlandicus*, like other insect species, might differ from the temperatures measured in the shade due to behavioral thermoregulation (e.g., Stevenson, 1985; Kearney et al., 2009), such as seeking microhabitat temperatures that deviate from air temperatures (Stevenson, 1985; Böcher and Nachman, 2001; Danks, 2004; Kearney et al., 2009). However, as sampled individuals are collected in the same microhabitat as where air temperatures are measured, we do argue that the difference between air and body temperatures is likely to be minor. The adult life-stage of *N. groenlandicus* seemingly has a high preferred body temperature of approximately 30°C (Böcher and Nachman, 2001) and an extreme thermal tolerance breadth spanning from critical lower limits (CTmin) of −3.4°C to critical upper thermal limits (CTmax) of 48.5–52°C (Böcher and Nachman, 2001; Sørensen et al., 2019; Bahrndorff et al., 2021b). Thus, only the lowest temperatures recorded in the field approximated sub-lethal conditions for the species. Typically, hardening responses are described as being induced by stressful conditions (Angilletta, 2009). For example it has been described from laboratory and field studies performed on *Drosophila* spp. that hardening temperatures ca. 10–15°C above optimal rearing temperatures are needed to induce adaptive increases in heat tolerance, and cause upregulation of heat shock proteins (Sørensen et al., 2003; King and MacRae, 2015). Here, despite exposure to temperatures well within their thermal comfort zone, we show that heat and cold tolerance changed daily in *N. groenlandicus*. This finding might represent an evolutionary adaptation to the extreme climatic variations of arctic environments, but may also suggest that temperature variation act in concert with changes in air humidity and/or other climate variables to affect thermal tolerances as found for several other polar and sub-polar species (Block et al., 1994; Hodkinson et al., 1996; Benoit et al., 2009; Everatt et al., 2015).

Another important discovery was that the patterns of plastic changes in cold tolerance were similar for males and females, while distinct patterns were seen for heat tolerance for the two sexes (Figure 2). Females had a higher HKDT (+5 min HKDT) compared to males and tended to exhibit a stronger plasticity for that trait when field temperatures varied. Often, studies on thermal plasticity in insects find that the variation in upper thermal limits is constrained, and less plastic, compared to lower thermal limits (reviewed by Hoffmann et al., 2013; see also Chown, 2001; Overgaard et al., 2011; Alford et al., 2012). This is likely resulting from the difficulty of insects to seek shelter from cold temperatures, thus resulting in a stronger selection pressure for plasticity of cold tolerance (Hoffmann et al., 2013). Our findings suggest that the selection pressure for cold tolerance may have been similar in male and female *N. groenlandicus* because no differences were observed in T_recovery_. Conversely, heat stress is often countered by behavioral thermoregulation in ectotherms, for instance by seeking shadow or migrating below-ground (Huey and Tewksbury, 2009; Kearney et al., 2009). The higher heat tolerance and plasticity for this trait in females could be explained by the univoltine life history and the short arctic summers requiring females to seek out warm temperatures to rapidly complete their life cycle (Bahrndorff et al., 2021a). Further, our results indicate a trade-off between heat and cold tolerance. Thus, individuals sampled at middays and afternoons are overall more heat tolerant and less cold tolerant compared to individuals sampled during mornings and evenings. Similar results have been found for thermal tolerance of the fruit fly *Drosophila melanogaster* kept under natural and semi-natural conditions (Overgaard and Sørensen, 2008; Schou et al., 2015). A consequence might be maladaptive plastic responses to environmental cues, as climatic conditions are prospected to become more unpredictably variable in the future (Kingsolver and Huey, 1998; Huey et al., 1999; Manenti et al., 2014).

### Daily Thermal Variations and Metabolic Fingerprints

The separation of the metabolic fingerprints from field-sampled *N. groenlandicus* was much stronger when individuals were collected during the thermally variable day (day 5), as compared with the less temperature variable day (day 4). This finding supports our hypothesis that microhabitat environmental conditions have a strong impact on diurnal changes of the physiology of adult *N. groenlandicus*. It also suggests that the observed diurnal variation in metabolic fingerprints cannot be explained by circadian clock regulations alone because similar patterns would be expected on the two collection days if that was the case.

On the less variable day, the average temperature prior to testing the insects was rather similar for the four collection periods (maximal temperature difference of 4.9°C; Figure 1). The measured changes in metabolite concentrations on this day were thus mostly independent of temperature, and rather reflected adjustments in energetic metabolism over the day or circadian regulated responses independent of temperature. This assumption is supported by the grouping pattern of the metabolomic profiles of individuals collected at the four different time points of day 4. Metabolomic profiles in the morning and evening were more similar and separated from those of individuals collected on the midday and afternoon. This pattern may reflect that the activity of the individuals was higher during midday and afternoon and in turn increased the energetic needs and metabolism in general. Consistently, sugars (glucose, fructose, galactose), some metabolic intermediates (citric and fumaric acid), and a range of free amino acids (phenylalanine, valine, serine, glutamine, and tyrosine), all being important substrates for glycolysis and Krebs cycle, varied in rhythmic patterns on both day 4 and 5 (Figure 3). These patterns might constitute circadian clock mechanisms that are regulated independently of temperature, humidity and other variable abiotic factors as seen, e.g., in *D. melanogaster* (Rhoades et al., 2018).

On the temperature variable day, the pattern of separation was markedly different than the one reported for day 4. The groups separating the strongest and explaining most of the variation in the data belonged to the individuals collected in the morning and afternoon, representing the time points with the lowest and the highest temperatures of day 5. Throughout this day, the sugars fructose, glucose and galactose, occurred in higher quantities compared to day 4 and especially fructose and glucose accumulated 2–3 fold in the evening in the individuals sampled on day 5, despite the temperature not being different from the temperature on day 4 at this time point (Figures 1, 3). Typically, sugar accumulation is associated with cold shock responses (Jagdale et al., 2005; Lalouette et al., 2007; Michaud and Denlinger, 2007; Overgaard et al., 2007; Holmstrup et al., 2010; Teets et al., 2011) and seasonal preparation for diapause (Koštál et al., 2001; Watanabe, 2002; Vasquez et al., 2019). Sugars have osmoprotective properties that may play important protective roles in cold tolerance possibly through stabilization of cell membranes and macromolecular structures even at low concentration (Gekko and Timasheff, 1981; Yancey, 2005; Koštál et al., 2016) or by maintaining haemolymph osmolality despite low [Na+] and [K+] due to cold exposure (MacMillan et al., 2015). Thus, sugars might be accountable for the higher cold tolerance observed in individuals on days characterized by large temperature amplitude.

Polyols accumulated during the warmest periods of the days and especially inositol, fluctuated on day 5. Accumulation of the polyols mannitol and sorbitol in whiteflies and aphids with daily warm peaks has been associated with increased heat tolerance under natural and semi-natural conditions (Hendrix and Salvucci, 1998; Wolfe et al., 1998; Salvucci et al., 2000). This could indicate that polyols contribute to regulation of heat tolerance in *N. groenlandicus*.

It is possible that oscillations of sugars and polyols were affected by temperature-dependent activity patterns such as feeding, mating and general metabolism, and this might confound the effects of temperature and humidity alone on thermal tolerance. Foraging or feeding rates are partly governed by upper and lower activity thresholds of organisms (Everatt et al., 2013). Our own unpublished data on the activity of *N. groenlandicus* show that the species is virtually inactive at temperatures below 15°C and activity peaks at 35–40°C. This might suggest that feeding is constricted to the warmest periods of the day (typically between 20 and 30°C at the given study site). *Nysius* species feed on phloem sap and plant seeds (Böcher, 1972; Broadle et al., 1986; Böcher et al., 2015; Tiwari and Wratten, 2019; Maharjan et al., 2020), and therefore ingest large quantities of sucrose, which is produced by photosynthesis in plants. In other hemipteran phloem-feeders, sucrose is hydrolyzed to glucose and fructose when ingested and rapidly converted to trehalose or polyols, which are less toxic compounds to store in the hemolymph at high concentrations (Becker et al., 1996; Hendrix and Salvucci, 1998). Thus, there might be a direct link between feeding behavior and sugar and polyol levels. For instance, trehalose concentrations increased more in the afternoon/evening on the warm day when feeding rates are expected to be higher (Figure 3).

Additionally, selective feeding on protein- and lipid rich diets impact on thermal tolerance in several insect species. For instance, the dung beetle *Thorectes lusitanicus* has been found to selectively supplement its diet with acorn, and experiments showed that beetles that were fed on acorn had a hemolymph supercooling point that was 5°C lower than individuals fed on cow-dung (Verdú et al., 2010). The shift was associated with alterations in hemolymph cryoprotectant content. Likewise, Rho and Lee (2017) showed that the beetle *Tenebrio molitor* selectively chose a carbohydrate rich diet at cold and warm temperatures, opposed to a more balanced protein-carbohydrate diet at intermediate temperatures. Switches in feeding preference with cold stress have also been found for *Drosophila* species (Brankatschk et al., 2018; Strassburger and Teleman, 2018). These aspects should be examined further in future studies.

Whether the oscillations of sugars and polyols constitute protective responses rather than consequences of altered energetic metabolism or temperature-dependent feeding is not evident from our results. However, the contrasting patterns of sugar and polyol oscillation may indicate that polyols are converted to sugars during the coldest times of the thermally variable day, maybe as a protective response. It might also explain the negative trade-off observed between heat and cold tolerance in this study. However, this is speculative and should be examined further in future studies along with common garden experiments revealing the importance of circadian rhythm regulation of *N. groenlandicus* behavior and physiology.

## Conclusion

Here, we showed that thermal tolerance was correlated with ambient microhabitat temperature in the Greenlandic seed bug, *N. groenlandicus*. Thus, we show that hardening responses observed under constant laboratory conditions in a previous study (Sørensen et al., 2019) occur also in field settings for this species. Interestingly, we found that the plastic adjustments of thermal tolerance occurred at relative benign temperatures contrasting experimental evidence from laboratory studies on primarily model species, suggesting that these responses occur at sub- or supra lethal temperatures. Thus, plasticity of thermal tolerance is likely affected by multiple factors under natural conditions and constitute an example of evolutionary adaption to the extreme and variable Arctic and sub-Arctic habitats of *N. groenlandicus*. Further, we showed that field heat hardening causes increased heat tolerance and reduced cold tolerance and *vice versa* with cold acclimation, suggesting a trade-off. GC-MS investigation of field collected individuals revealed candidate metabolites that are regulated according to thermal and other abiotic and biotic conditions that vary on a diurnal basis. Distinct metabolic fingerprints associated with temperature on the thermally variable day (day 5) were not observed at the thermal stable day (day 4). This suggests that our results cannot be explained by circadian clock regulated mechanisms alone, but that these metabolites are partly regulated by temperature variability (or variation in correlated environmental or physiological variables) and constitute important physiological mechanisms controlling diurnal variability in thermal tolerances in the species. The strong plastic responses observed in heat and cold tolerance and in the metabolomic profiles in our study suggests ongoing strong selection for plasticity in this highly fluctuating polar environment. The genetic architecture of these traits are mainly investigated in model organisms where studies on *D. melanogaster* suggest significant heritable variation for plasticity of thermal stress tolerance traits and metabolite profiles (Gerken et al., 2015; Hangartner and Hoffmann, 2015; Ørsted et al., 2017; Rohde et al., 2021).

## Data Availability Statement

All data, including raw field temperature files and GC-MS data, are presented in this article/Supplementary Material.

## Author Contributions

SB, TNK, and NKN conceived the ideas and designed the methodology. MHS, SB, TNK, and NKN collected the data. DR, HC, and NKN processed samples for metabolomics analysis and analyzed the metabolomics data. TNK and NKN led the writing of the manuscript. All authors contributed to the drafts and gave final approval for publication.

## Conflict of Interest

The authors declare that the research was conducted in the absence of any commercial or financial relationships that could be construed as a potential conflict of interest.

## Publisher’s Note

All claims expressed in this article are solely those of the authors and do not necessarily represent those of their affiliated organizations, or those of the publisher, the editors and the reviewers. Any product that may be evaluated in this article, or claim that may be made by its manufacturer, is not guaranteed or endorsed by the publisher.
