# Supplementary material for: Rapid Adjustments in Thermal Tolerance and the Metabolome to Daily Environmental Changes – A Field Study on the Arctic Seed Bug Nysius groenlandicus
Source: Front Physiol. 2022 Feb 16;13:818485. doi: 10.3389/fphys.2022.818485 (PMC8889080; doi:10.3389/fphys.2022.818485)
Supplement: Supplementary file 1 [file Data_Sheet_1.docx]

Supplementary Material

**
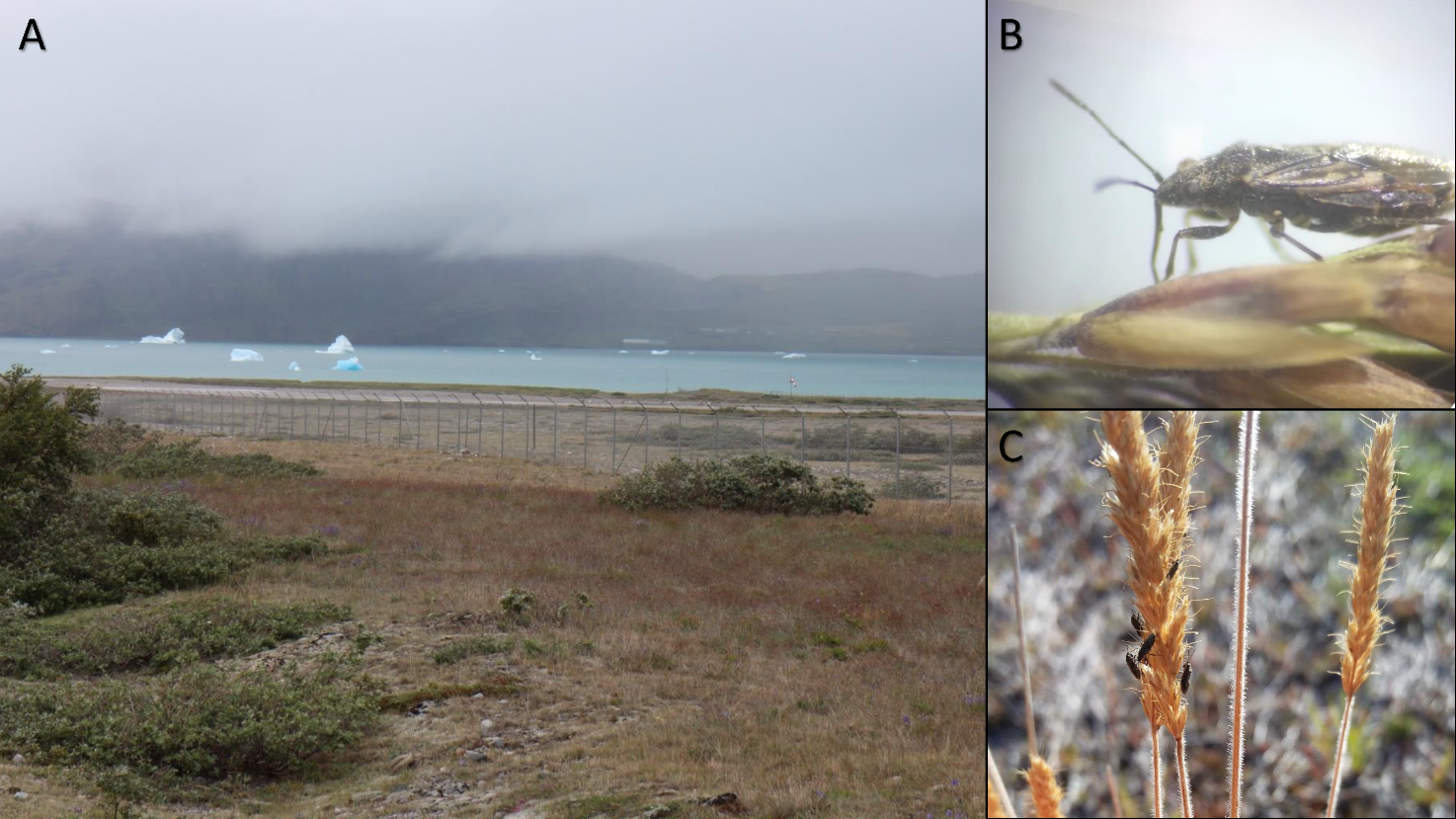
**

**Figure S1:** The fieldwork site (A) is a grass-covered and dry habitat located along the bank of Tunulliarfik Fjord. Adult *N. groenlandicus* was collected from the grasses (B and C) using a sweep net.

**Table S1:** Overview of dates and times for field collection of *N. groenlandicus*, start times of thermal assays (heat knockdown time (HKDT) and chill coma recovery temperature (T_recovery_)), and microhabitat temperature summary. The insects were kept in glass containers in the shadow in the field site until assay start. Stars (*) indicate time points when samples for GC-MS analysis were collected simultaneously with individuals for thermal tolerance tests. Microhabitat temperatures were summarized by average, minimum, maximum, and daily range (max-min) in the 1 hour timespan prior to assay start.

|  | Sampling date | HKDT assay start | T_recovery_ assay start | Avr | Min | Max | Range |
| --- | --- | --- | --- | --- | --- | --- | --- |
| Day 1 | 07-08-2018 | 08:30 | 08:28 | 9.4 | 7.0 | 11.5 | 4.5 |
|  | 07-08-2018 | 12:10 | 12:09 | 25.3 | 22.0 | 28.0 | 6.0 |
|  | 07-08-2018 | 16:08 | 16:06 | 24.5 | 23.5 | 26.0 | 2.5 |
|  | 07-08-2018 | 19:58 | 19:58 | 17.1 | 15.0 | 19.5 | 4.5 |
| Day 2 | 10-08-2018 | 08:22 | 08:14 | 10.7 | 10.0 | 12.0 | 2.0 |
|  | 10-08-2018 | 12:20 | 12:15 | 26.3 | 23.0 | 28.5 | 5.5 |
|  | 10-08-2018 | 17:08 | 17:09 | 23.6 | 22.0 | 24.5 | 2.5 |
|  | 10-08-2018 | 20:35 | 20:32 | 15.9 | 14.5 | 17.0 | 2.5 |
| Day 3 | 11-08-2018 | 08:14 | 08:08 | 10.1 | 8.5 | 11.0 | 2.5 |
|  | 11-08-2018 | 12:15 | 12:09 | 18.0 | 17.5 | 19.0 | 1.5 |
|  | 11-08-2018 | 16:16 | 16:09 | 21.5 | 20.0 | 24.0 | 4.0 |
|  | 11-08-2018 | 20:20 | 20:11 | 12.5 | 12.0 | 13.5 | 1.5 |
| Day 4 | 22-08-2018* | 08:51 | 08:44 | 8.1 | 7.0 | 8.5 | 1.5 |
|  | 22-08-2018* | 12:22 | 12:15 | 10.2 | 10.0 | 10.5 | 0.5 |
|  | 22-08-2018* | 16:22 | 16:15 | 13.0 | 11.5 | 14.5 | 3.0 |
|  | 22-08-2018* | 20:20 | 20:15 | 8.8 | 7.0 | 10.0 | 3.0 |
| Day 5 | 27-08-2018* | 08:20 | 08:13 | 1.0 | 0.5 | 2.5 | 2.0 |
|  | 27-08-2018* | 12:39 | 12:33 | 22.2 | 18.0 | 25.0 | 7.0 |
|  | 27-08-2018* | 16:29 | 15:25 | 16.0 | 13.5 | 21.5 | 8.0 |
|  | 27-08-2018* | 20:25 | 16:21 | 8.6 | 7.0 | 10.0 | 3.0 |


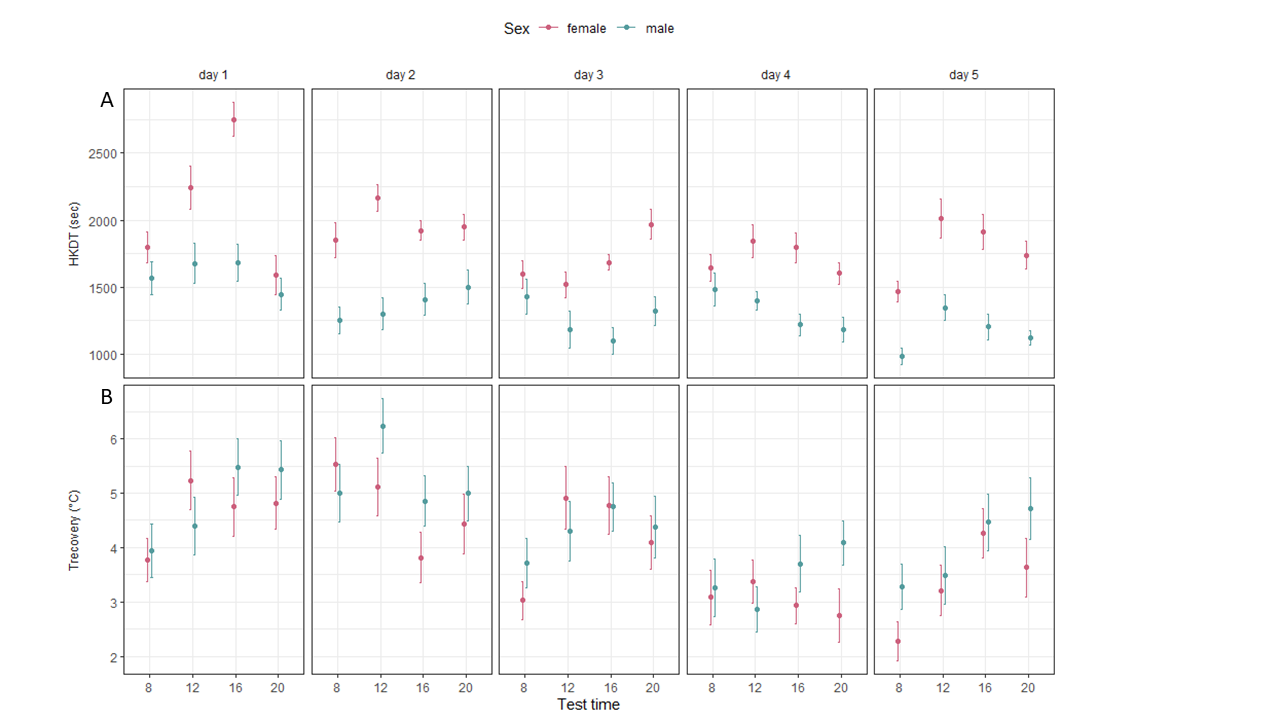


**Figure S2:** A) Mean heat knockdown time (seconds) and B) chill coma recovery temperature (°C) for female (red) and male (blue) *N. groenlandicus* collected across different times and days. Bars are standard errors of the mean.

**Table S2:** Summary of two-way ANOVAs on rank inverse transformed HKDT and T_recovery_ of individuals (n=20 pr assay) as dependent variables and ‘day’ and ‘Time of day’ as independent variables.

|  | **ANOVA Heat knockdown time** | | | | | |  |
| --- | --- | --- | --- | --- | --- | --- | --- |
| sex | variable | Df | Sum sq | mean sq | F value | Pr(>F) |  |
| females | day | 4 | 27.25 | 6.81 | 8.62 | 1.14E-06 | *** |
|  | time | 3 | 23.37 | 7.79 | 9.86 | 2.83E-06 | *** |
|  | day:time | 12 | 47.61 | 3.97 | 5.02 | 9.15E-08 | *** |
|  | Residuals | 379 | 299.47 | 0.79 |  |  |  |
|  |  |  |  |  |  |  |  |
| males | day | 4 | 28.30 | 7.08 | 7.70 | 5.65E-06 | *** |
|  | time | 3 | 0.70 | 0.24 | 0.26 | 0.855 |  |
|  | day:time | 12 | 20.00 | 1.67 | 1.82 | 0.044 | * |
|  | Residuals | 379 | 348.60 | 0.92 |  |  |  |
|  |  |  |  |  |  |  |  |
|  | **ANOVA chill coma recovery temperature** | | | | | |  |
| females | day | 4 | 40.80 | 10.21 | 11.77 | 5.10E-09 | *** |
|  | time | 3 | 9.20 | 3.06 | 3.52 | 0.015 | * |
|  | day:time | 12 | 18.90 | 1.57 | 1.81 | 0.045 | * |
|  | Residuals | 378 | 327.80 | 0.87 |  |  |  |
|  |  |  |  |  |  |  |  |
| males | day | 4 | 32.50 | 8.13 | 9.07 | 5.22E-07 | *** |
|  | time | 3 | 12.20 | 4.06 | 4.53 | 0.004 | ** |
|  | day:time | 12 | 13.60 | 1.13 | 1.26 | 0.238 |  |
|  | Residuals | 379 | 339.40 | 0.90 |  |  |  |

**Table S3:** Metabolites detected by GC-MS on whole-body extract on female *N. groenlandicus*. A total of 33 metabolites were detected. Two metabolites (marked by *) occurred at concentrations below the quantification limit.

| ***Free amino acids*** | ***Sugars*** | ***Polyols*** | ***Metabolic intermediates*** | ***Other metabolites*** |  |
| --- | --- | --- | --- | --- | --- |
| Alanine (Ala) | Fructose (Fru) | Adonitol | Citric acid | Citrulline |  |
| Isoleucine (Ile) | Galactose (Gal) | Arabitol | Fumaric acid | Ethanolamine* | |
| Leucine (Leu) | Glucose (Glc) | Inositol | Glyceric acid | Phosphoric Acid |  |
| Lysine (Lys) | Glucose-6-phosphate (G6P) | Glycerol | Lactic acid |  |  |
| Glutamic acid (Glu) | Trehalose (Tre) | Glycerol-3-Phosphate | Malic acid |  |  |
| Glycine (Gly) |  | Xylitol* | Succinic acid |  |  |
| Ornithine (Orn) |  |  |  |  |  |
| Phenylalanine (Phe) |  |  |  |  |  |
| Proline (Pro) |  |  |  |  |  |
| Serine (Ser) |  |  |  |  |  |
| Threonine (Thr) |  |  |  |  |  |
| Tyrosine (Tyr) |  |  |  |  |  |
| Valine (Val) |  |  |  |  |  |

**Table S4:** Raw data of detected metabolite concentrations from *N. groenlandicus* whole-body extract by GC-MS. Concentrations (nmol.mg-1) are listed for each metabolite (columns) and each replicate (rows) of the different sampling times and days (Excel).

**Table S5:** Results from one-way Analysis of Variance (ANOVA) performed on log-transformed metabolite data for day 4 and day 5 separately. The dependence of sampling time (morning, midday, afternoon, and evening) on metabolite concentration was examined for each metabolite (Excel).


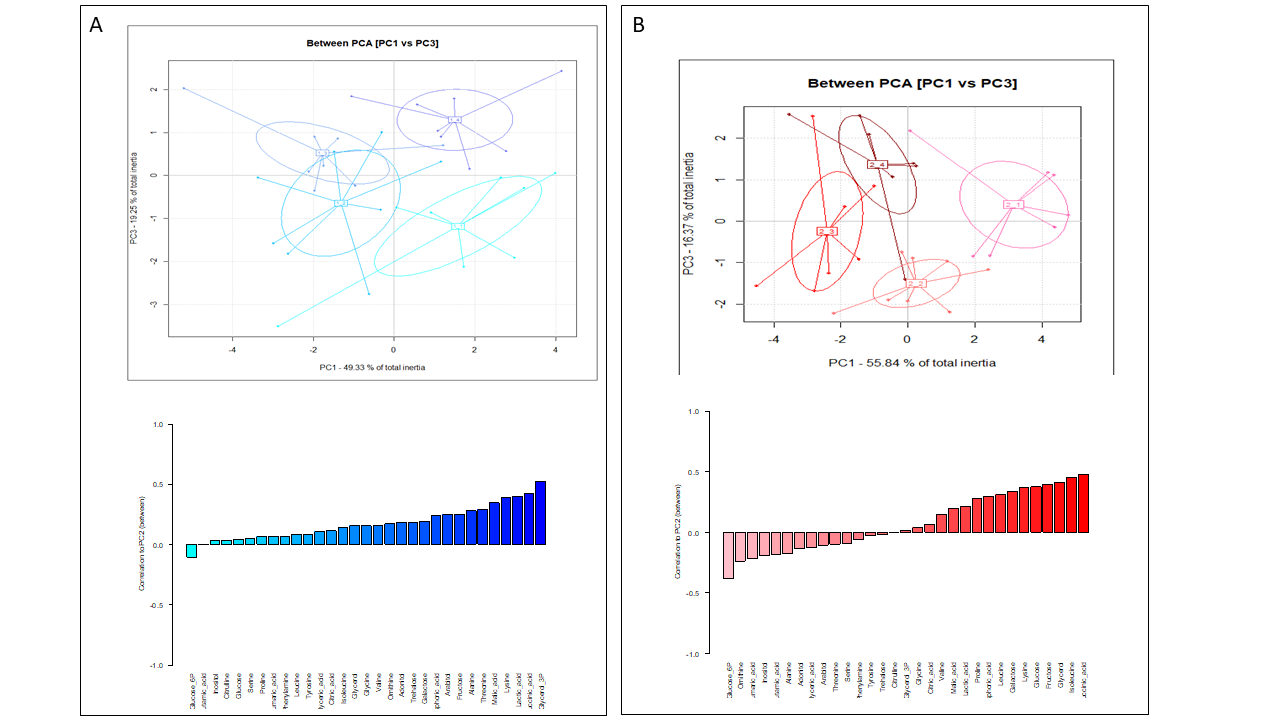


**Figure S3:** Between-class Principal Component Analyses (PCA) based on GC-MS on whole-body extracts of female *N. groenlandicus* sampled in the field at four consecutive sampling time points (8:00 am, 12:00 pm, 4:00 pm, 8:00 pm) on **A)** day 4 and **B)** day 5. Scores for PC1 and PC3 are depicted. Lines represents individual sample position respective to centroids (n=8). Correlations of metabolite concentrations (relative proportions) to PCs in the between-class PCA. A and B represent metabolite correlations to PC2 for day 4 and day 5, respectively.
